# Focal-type, but not Diffuse-type, Amyloid Beta Plaques are Correlated with Alzheimer’s Neuropathology, Cognitive Dysfunction, and Neuroinflammation in the Human Hippocampus

**DOI:** 10.1007/s12264-022-00927-5

**Published:** 2022-08-26

**Authors:** Fan Liu, Jianru Sun, Xue Wang, Sixuan Jin, Fengrun Sun, Tao Wang, Bo Yuan, Wenying Qiu, Chao Ma

**Affiliations:** 1 National Human Brain Bank for Development and Function, School of Basic Medicine Peking Union Medical College, Institute of Basic Medical Sciences Chinese Academy of Medical Sciences, Beijing, 100005 China; 2 Department of Human Anatomy, Histology and Embryology, Neuroscience Center, School of Basic Medicine Peking Union Medical College, Institute of Basic Medical Sciences Chinese Academy of Medical Sciences, Beijing, 100005 China; 3 Chinese Institute for Brain Research, Beijing, 102206 China

**Keywords:** Focal Aβ plaques, ECog score, ABC score, Alzheimer’s disease, Neuroinflammation, Clinicopathological correlation, Human brain bank

## Abstract

Amyloid beta (Aβ) plaques are one of the hallmarks of Alzheimer’s disease (AD). However, currently available anti-amyloid therapies fail to show effectiveness in the treatment of AD in humans. It has been found that there are different types of Aβ plaque (diffuse and focal types) in the postmortem human brain. In this study, we aimed to investigate the correlations among different types of Aβ plaque and AD-related neuropathological and cognitive changes based on a postmortem human brain bank in China. The results indicated that focal plaques, but not diffuse plaques, significantly increased with age in the human hippocampus. We also found that the number of focal plaques was positively correlated with the severity of AD-related neuropathological changes (measured by the “ABC” scoring system) and cognitive decline (measured by the Everyday Cognitive Insider Questionnaire). Furthermore, most of the focal plaques were co-localized with neuritic plaques (identified by Bielschowsky silver staining) and accompanied by microglial and other inflammatory cells. Our findings suggest the potential of using focal-type but not general Aβ plaques as biomarkers for the neuropathological evaluation of AD.

**Supplementary Information:**

The online version contains supplementary material available at 10.1007/s12264-022-00927-5.

## Introduction

Alzheimer’s disease (AD) causes prolonged suffering in patients and societal issues. Amyloid-beta (Aβ) deposits, neurofibrillary tangles (NFTs), and neuritic plaques (NPs) are the hallmark pathologic findings in the brains of AD patients [1]. Aβ, produced by the sequential cleavage of β-amyloid precursor protein through β-secretase and γ-secretase, is released from neurons through both presynaptic and postsynaptic modulation [2–5]. NPs are pathological structures containing Aβ along with neural and microglial elements. Dystrophic neural processes containing aggregated, phosphorylated tau surrounding Aβ deposits are a key feature of NPs [6]. Toxic Aβ deposits evoke a cascade of oxidative damage-dependent apoptosis in neurons [7–9]. Earlier studies have shown that the accumulation of Aβ plaques enhances the formation of NFTs and NPs, and this may be necessary for the progression of tau pathology in AD [10–12]. Although the amyloid cascade hypothesis seems to be that Aβ plays a key role in AD [10, 13–15], until now, therapies involving anti-Aβ treatments, including Aβ-directed monoclonal antibodies, Aβ vaccines, Aβ aggregation inhibitors and β-secretase inhibitors, have failed in clinical trials [16, 17].

Current anti-Aβ treatments for AD are poorly effective, owing to limited knowledge of the pathophysiological mechanisms underlying Aβ [10, 17–20]. Some studies have reported a lack of correlation between Aβ plaques and cognitive decline in AD patients [20–22]. Based on the postmortem neuropathological diagnosis of AD, some studies have found that Aβ is also widely present in the elderly human brain with normal cognition [23]. PET imaging studies found small amounts of Aβ deposition in the brains of some diagnosed AD patients [24, 25]. Recently, more studies have suggested that Aβ has multiple physiological functions, such as the regulation of learning and memory, angiogenesis, and neurogenesis, and the promotion of recovery from injury [18, 19].

Aβ peptides of different lengths can transform into oligomeric and fibril forms and eventually form amyloid plaques [2, 18, 19]. Much research has strongly suggested that Aβ, ranging from soluble oligomers to insoluble fibrils and plaques, causes and leads to the pathogenic cascade of AD [26]. The Aβ42 peptide has been found to be more neurotoxic and more prone to aggregation than the Aβ40 peptide [27, 28]. Many studies have also reported that Aβ oligomers are more neurotoxic than plaques and can lead to cognitive deficits [29]. Based on postmortem neuropathology of the human brain, Aβ plaques have different morphological forms, which are mainly classified into diffuse and focal (or dense-core) types [30–32]. Diffuse Aβ plaques are seen on immunostaining as loose structures with irregular, ill-defined margins, while focal Aβ plaques have clear-cut outlines and generally have a core [32]. Although electron microscopic analysis of the postmortem human brain has shown that all forms of Aβ plaque are associated with neuropathology, there are significant differences between diffuse and focal Aβ plaques [33, 34]. Previous studies revealed that focal Aβ plaques may cause neuronal damage and microglial activation, which are closely associated with the pathological changes of AD [30, 31].

AD patients undergo progressive Aβ plaque deposition followed by surrounding neuritic and glial cytopathology in the brain [31, 35]. The accumulation of toxic forms of Aβ affects synaptic function and plasticity and triggers glial activation and neuroinflammation [35]. However, it is not fully clear which specific Aβ plaque type is involved. Here, we explore the correlation among different Aβ plaque forms, brain pathology, clinical cognition, and neuroinflammation based on cases from the National Human Brain Bank for Development and Function, Institute of Basic Medical Sciences, Chinese Academy of Medical Sciences.

## Materials and Methods

### Human Brain Sample Sources

A total of 92 human brain samples fixed in 10% formalin from the National Human Brain Bank for Development and Function, Institute of Basic Medical Sciences, Chinese Academy of Medical Sciences, were used in this research. Detailed information, including demographic variables, ABC scores, ECog scores, and other information on all donors, are listed in Table S1. The research protocol was approved by the Institutional Review Board of the Institute of Basic Medical Sciences of the Chinese Academy of Medical Sciences, China (Approval Numbers: 009–2014 and 031–2017).

### Neuropathological Evaluation

According to the guidelines of the National Institute on Aging and Alzheimer’s Association (NIA-AA), all brain tissue received identical neuropathological analysis by the “ABC” score, which was performed by a brain bank professional. The ABC score incorporates amyloid-β (Aβ) deposits (A Score), neurofibrillary tangles (B Score), and neuritic plaques (C Score). The A score reflects the order of amyloid-β appearance in the brain in a tiered manner, but the specific plaque type is not distinguished [36]. The general ABC score is categorized into 4 grades of AD neuropathological change: none (N), low (L), intermediate (I), and high (H). A neuropathological AD score of N/L indicates that the donor is unlikely to have AD and can be regarded as a normal elderly person. A neuropathological AD score of I/H indicates that the donor is very likely to have AD and can be considered a sufficient explanation for dementia [36]. Among 92 Aβ-positive brain tissue donors, 19 had AD neuropathological scores that were L, and 73 were I/H, which were very likely to have AD. The detailed pathological information of each case is listed in Table S1.

### Cognitive Function Assessment

Of the 92 human brain samples, only 84 had ECog scores. Clinical cognitive status was determined using the Everyday Cognitive (ECog) Insider Questionnaire, which includes 39 questions aimed to assess the daily cognitive function of the brain donors. And there are 6 sub-items (memory, language, visuospatial functions, planning, organization, and divided attention) in the ECog questionnaire [37, 38]. In accordance with the criteria for ECog scores, normal cognition was defined as an ECog score ≤1.0, mild cognitive impairment as an ECog score 1.0–2.0, and dementia as an ECog score >2.0.

### Immunohistochemistry and Immunofluorescence Staining

Based on the NIA-AA guidelines, these brain regions (superior frontal cortex, primary motor cortex, inferior temporal cortex, hippocampus, anterior cingulate cortex, amygdala, supramarginal cortex, caudate/putamen, midbrain, pons, medulla oblongata, and cerebellar dentate nucleus) were sampled from postmortem human brains and brain tissues were embedded in paraffin. Given that Aβ plaques are stereoscopic and that the top view of a plaque would differ from the middle and bottom views, we applied serial section staining to compensate for this drawback. Specifically, we randomly selected one sample in which the ABC score was “I” and had a certain number of focal plaques and diffuse plaques. Then, five paraffin sections were cut at 5 µm continuously for Aβ immunohistochemical staining. We randomly selected 4 visual fields. For each section, we judged the subtype of each Aβ plaque in the 4 fields. The actual subtype of each Aβ plaque was judged by synthesizing five sections. Then, we calculated the accuracy of plaque judgment for each section to finally obtain the average accuracy. To study the co-localization of Aβ plaques and NPs, 5 human brain hippocampi covering the “L/I/H” group were serially sectioned, and the adjacent sections were subjected to Aβ immunohistochemical staining and modified Bielschowsky for NPs.

In staining for pathological A and B scoring, the paraffin-embedded brain samples were cut at 5 µm, and for C scoring at 10 µm. The staining for the A and B scores was based on immunohistochemistry against β-amyloid (mouse monoclonal antibody, diluted 1:200, DAKO Cat#M0872) and p-Tau (Ser202, Thr205) (p-Tau, mouse monoclonal antibody, 1:800, Thermo Cat#MN1020). The method for Aβ deposits was immunohistochemistry for Aβ, and for NFTs, the method was immunohistochemistry for p-Tau (Ser202, Thr205) [21]. The primary antibodies were incubated separately overnight at 4°C and then processed for 60 min with a mouse two-step detection kit (mouse enhanced polymer detection system; ZSGB-BIO PV-9002, Beijing, China). The staining results were visualized with a DAB chromogenic kit (ZSGB-BIO ZLI-9019, Beijing, China). The C score was determined by modified Bielschowsky staining for neuritic processes in senile plaques [39].

Formalin-fixed hippocampal tissue was embedded in OCT medium (Sakura) and then cut at 16 μm on a cryostat for immunofluorescent staining. Double immunofluorescence staining was used for co-labeling of Aβ and IBA1. For the co-labeling of Aβ and CD68, CD86, or CD19, the same section was used prior to Aβ immunohistochemistry, followed by immunofluorescence staining for the CD molecule. The frozen sections were incubated separately overnight at 4°C in primary antibodies (rabbit anti-IBA1, 1:200, Abcam Cat#ab178846; mouse anti-CD68, 1:1000, Abcam Cat#ab955; rabbit anti-CD86, 1:200, Cell Signaling Technology Cat#91882S; rabbit anti-CD19, 1:800, Cell Signaling Technology Cat#90176T; rabbit anti-Aβ, 1:200, Abcam Cat#ab2539; mouse anti-Aβ, 1:200, DAKO Cat#M0872) and then incubated with the appropriate secondary antibodies (Alexa Fluor 594-conjugated goat anti-rabbit, 1:400; Alexa Fluor 488-conjugated goat anti-mouse, 1:500; HRP-conjugated goat anti-mouse, 1:500; or HRP-conjugated goat anti-rabbit, 1:500). DAB reagent was added after incubation with HRP-conjugated secondary antibodies. The slides were then washed in PBS and cover-slipped with Vectashield mounting medium with DAPI. Images of the sections were captured using a microscopic imaging system (Olympus BX61 and FluoView software).

Immunohistochemistry against Aβ was analyzed in ten 10× microscopic fields using ImageScope software from a similar position in human brain hippocampus sections, including CA1-CA4, subiculum, and entorhinal cortex, and the actual tissue area observed was 20 mm^2^. The summation represented the number of Aβ plaques. We divided Aβ plaques into two types: diffuse and focal. Plaques looking like loose structures with irregular and ill-defined margins were defined as diffuse; and plaques that had clear-cut outlines and generally had a compact core or strong sepia granular texture were defined as focal. Then, the percentage of each plaque type can be calculated.

### Statistical Analysis

Values are expressed as the mean ± SEM. Statistical analyses were conducted using SPSS software (version 17.0) and GraphPad software. Student’s *t* test was used to analyze the statistical significance of differences between 2 groups, and one-way analysis of variance (ANOVA) followed by Scheffe’s *post-hoc* test was used to analyze the statistical significance of differences among 3 groups. The relationship between different types of data was analyzed with Spearman correlation analysis. *P* <0.05 was considered significant.

## Results

### Association Between Hippocampal Aβ Plaque Forms and Demographic Variables

**Fig. 1:**
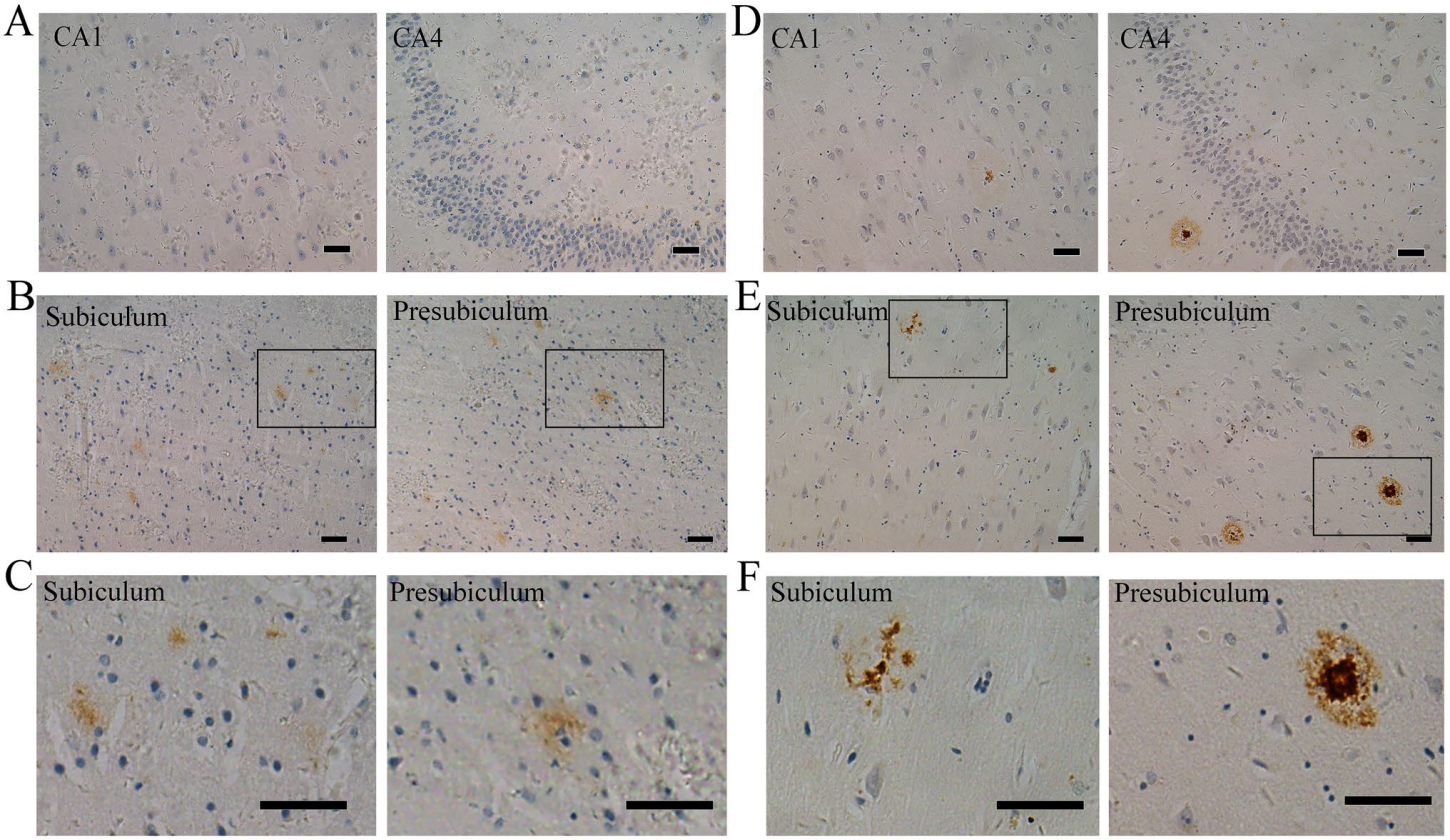
Histochemical staining showing the morphology of different forms of Aβ plaque in the human hippocampus. **A, B** Diffuse Aβ plaques in Aβ plaque-positive hippocampal tissue. Negative Aβ plaques in CA1 and CA4 (**A**) and positive diffuse Aβ plaques in the subiculum and presubiculum **(B)** with an ABC score of “L”. **C** Enlarged image of diffuse Aβ plaques in the subiculum and presubiculum. **D, E** Focal Aβ plaques from Aβ plaque-positive hippocampal tissue. Positive focal Aβ plaques in CA1–CA4 (**D**), subiculum and presubiculum (**E**) with an ABC score of “H”. **F** Enlarged image of focal Aβ plaques in the subiculum and presubiculum. Scale bars, 50 μm in (**A**–**F**).

IHC results revealed that hippocampal Aβ plaques existed in different forms in Aβ plaque-positive human brain samples (Fig. 1). The two most common subtypes, diffuse and focal Aβ plaques, were classified according to their unique appearance in hippocampal tissue. Diffuse Aβ plaques were seen on immunostaining as loose structures with irregular, ill-defined margins, while focal Aβ plaques had clear-cut outlines and generally had a core. Focal Aβ plaques are extracellular proteinaceous deposits composed of Aβ peptide, largely as amyloid filaments, while in diffuse plaques, the majority of the protein is not aggregated as amyloid filaments [32, 40]. It is important to further comprehend the differences between focal and diffuse plaques. Representative images of diffuse Aβ plaques in hippocampal tissue from samples with an “L” ABC score are shown in Fig. 1A–C. Representative images of focal Aβ plaques in the hippocampus from samples with an “H” ABC score are shown in Fig. 1D–F. Given that Aβ plaques are stereoscopic, we used serial section staining to calculate the accuracy of morphological judgment through only one section. Through the overall interpretation of 5 consecutive sections, we concluded that there were 10 diffuse plaques and 10 focal plaques. The average accuracy of random sections was 89% (Fig. S1A–E).

**Fig. 2:**
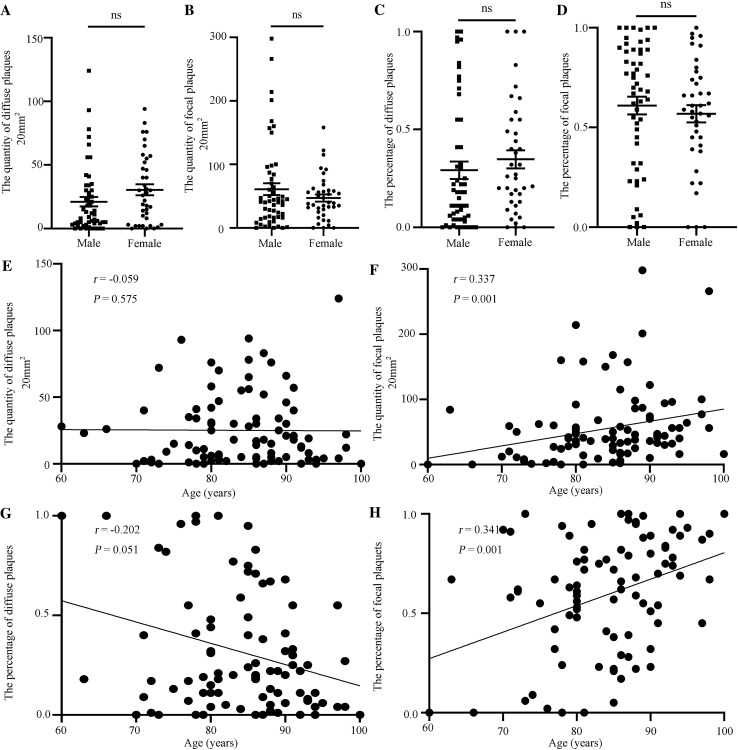
Correlation between demographic variables and Aβ plaque forms. **A** Quantification of diffuse Aβ plaques by gender. **B** Quantification of focal Aβ plaques by sex. **C** Percentage of diffuse Aβ plaques by sex. **D** Percentage of focal Aβ plaques by sex. In (**A**–**D)**, male = 53 samples, female = 39 samples, male group *versus* female group; ns, no significant difference, Student’s *t* test. **E** Quantification of diffuse Aβ plaques at different ages. *P* >0.05, no significant correlation by Spearman correlation. **F** Quantification of focal Aβ plaques at different ages. *P* = 0.001, *r* = 0.337, significant positive correlation by Spearman correlation. **G** Percentage of diffuse Aβ plaques at different ages. *P* >0.05, no significant correlation by Spearman correlation. **H** Percentage of focal Aβ plaques at different ages. *P* = 0.001, *r* = 0.341, significant positive correlation by Spearman correlation.

The association of demographic variables with the total number of Aβ plaques in the hippocampus was investigated. There was no significant difference in the total number by sex (Student’s *t* test, *P* >0.05) (Fig. S2A) or among the different age groups (one-way ANOVA, *P* >0.05) (Fig. S2C). The association of demographic variables with different forms of hippocampal Aβ plaque was investigated. There was no significant difference in the numbers or percentages of diffuse or focal Aβ plaques by sex (Student’s* t* test, *P* >0.05) (Fig. 2A–D). According to the age at death, Spearman rank correlation analysis showed no significant correlation between the number and percentage of diffuse plaques and age (Fig. 2E, G). The number and percentage of focal plaques showed a significantly positive correlation with age (Fig. 2F, H). The human brain mass was also used to investigate the correlation between Aβ plaques and brain atrophy. The results showed that the total number of Aβ plaques in the hippocampus was not significantly correlated with brain mass (Fig. S3A). Neither the number of diffuse nor of focal plaques showed significant correlation with brain mass (Fig. S3B–C). This suggests that as age increases, focal Aβ plaques in the human hippocampus tend to increase.

### Correlation Between Hippocampal Aβ Plaque Forms and AD-related Neuropathological Changes

**Fig. 3:**
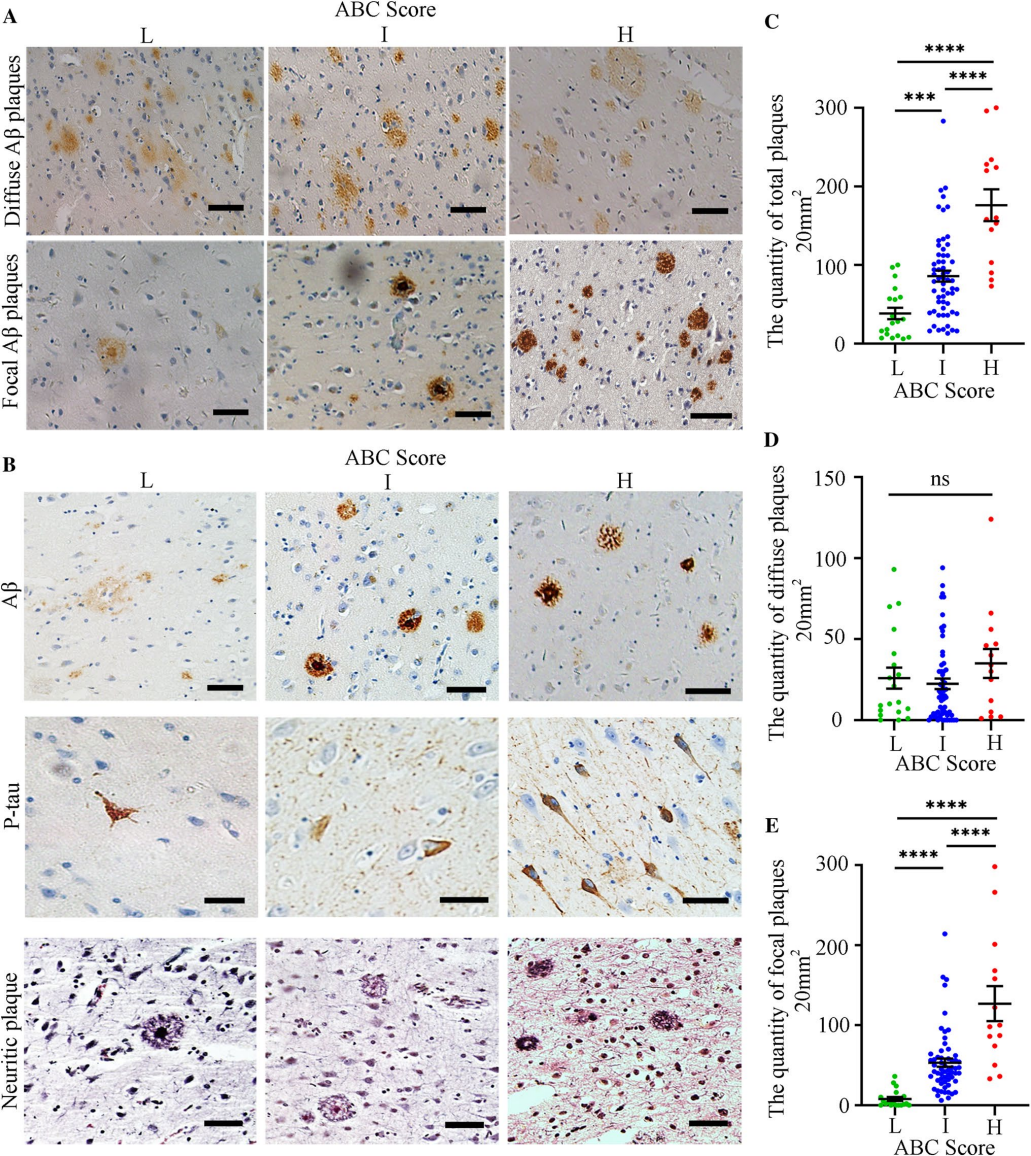
The number of hippocampal focal Aβ plaques increases with the degree of AD neuropathological change. **A** Distribution of diffuse and focal Aβ plaques in the hippocampus with different ABC scores of the human brain. **B** Neuropathological assessment of ABC score for Aβ plaque-positive human brain. In (**A**, **B**), scale bars, 50 μm. **C** The total number of Aβ plaques in the hippocampus from different ABC score groups. **D** Numbers of diffuse Aβ plaques in the hippocampus from different ABC score groups. **E** Numbers of focal Aβ plaques in the hippocampus from different ABC score groups. In (**C**–**E**), L group has 19 samples, I group has 59 samples, and H group has 14 samples, ***P <0.001, ****P <0.0001, ns, no significant difference by one-way ANOVA followed by Scheffe’s *post-hoc* test.

**Fig. 4:**
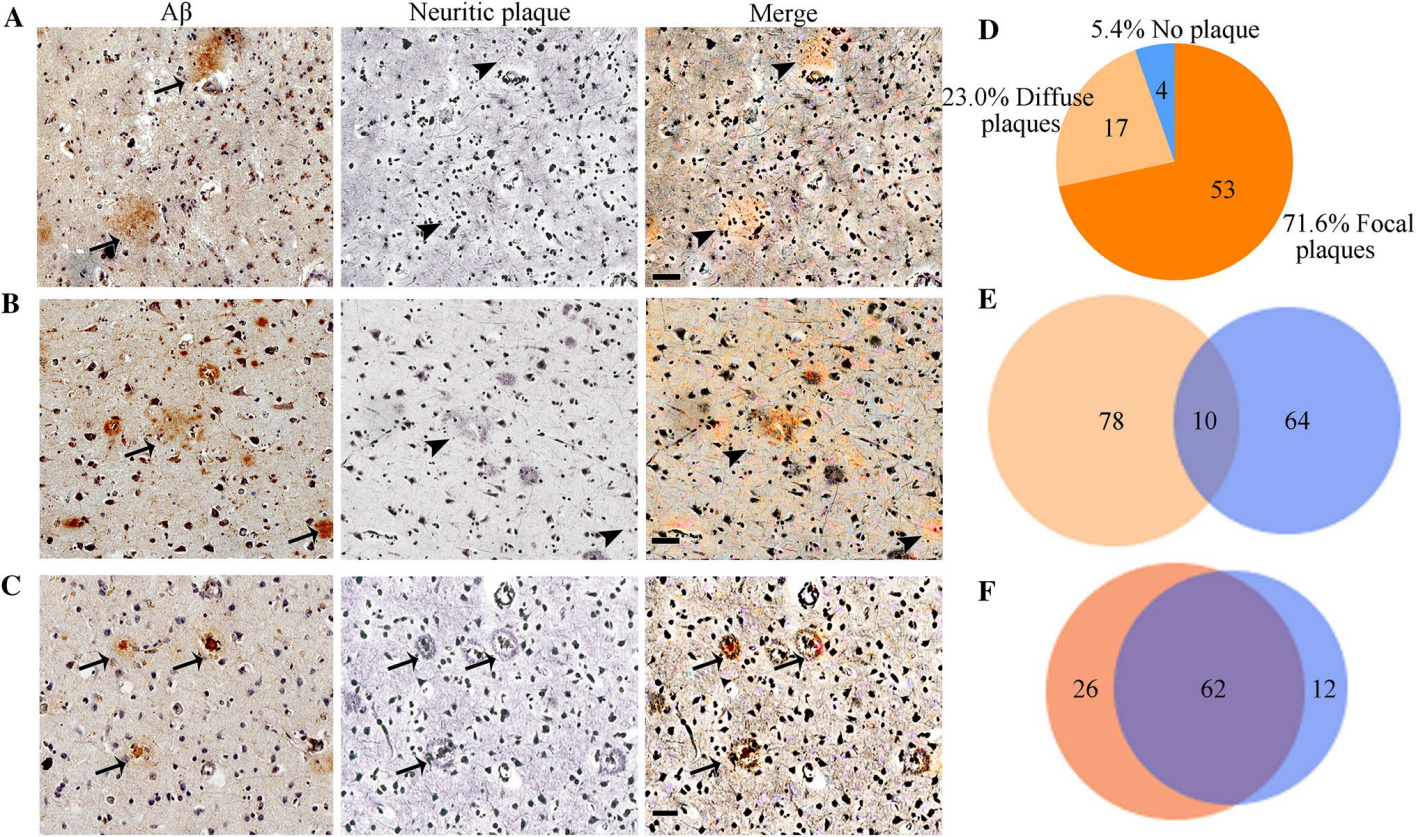
Correlation between different Aβ plaque forms and neuritic plaques in the hippocampus. **A** Histochemical staining of diffuse Aβ plaques and modified Bielschowsky staining of neuritic plaques in the hippocampus from the normal group. **B, C** Histochemical staining of diffuse (**B**) and focal (**C**) Aβ plaques and modified Bielschowsky staining of neuritic plaques in the hippocampus from the AD group. **D** The coincidence rate of neuritic plaques and different Aβ plaque forms. **E** The coincidence between diffuse Aβ plaques and neuritic plaques. A total of 11.4% of diffuse plaques coincided with neuritic plaques, and 13.5% of neuritic plaques coincided with diffuse plaques. **F** The coincidence between focal Aβ plaques and neuritic plaques. A total of 70.5% of focal plaques coincided with neuritic plaques, and 83.8% of neuritic plaques coincided with focal plaques. In (**A**–**C**), the arrows show Aβ plaques or neuritic plaques, and the arrowheads show no neuritic plaques. Scale bars, 50 μm. In (**E**–**F**), yellow represents Aβ plaques, and blue represents neuritic plaques.

IHC experiments indicated that the number of diffuse Aβ plaques did not significantly change in the hippocampus, while more focal Aβ plaques were observed in the intermediate (“I” ABC score) or high-level (“H” ABC score) AD neuropathological brain samples (Fig. 3A). The assessment of samples was conducted according to ABC scores, which included Aβ deposits, NFTs (p-Tau), and NPs. Fig. 3B shows immunohistochemical images of Aβ plaques, p-Tau, and NPs in groups with different ABC scores. One-way ANOVA revealed that the total number of Aβ plaques in the “I” and “H” score groups was significantly greater than that in the “L” group (Fig. 3C). Further analysis indicated that the number of focal plaques, but not diffuse plaques, was significantly different among the different ABC score groups in the hippocampus (Fig. 3D–E). The number of focal plaques in the “I” and “H” groups was significantly greater than that in the “L” group (Fig. 3E). The samples were serially sectioned, and adjacent sections were subjected to Aβ immunohistochemical staining and modified Bielschowsky for NPs. In the normal and AD group, diffuse plaques stained by immunohistochemistry and NPs stained by modified Bielschowsky showed almost no coincidence (Fig. 4A, B). However, the staining results showed that focal plaques coincided with NPs (Fig. 4C). We randomly counted 74 NPs from 5 hippocampi covering the “L/I/H” groups. The results showed that 71.6% of NPs coincided with focal Aβ plaques and 23.0% of NPs coincided with diffuse Aβ plaques (Fig. 4D). Randomly counting 88 diffuse plaques and 74 NPs from 5 hippocampi covering the “L/I/H” groups, showed only 11.4% diffuse plaques coincided with NPs and 13.5% of NPs coincided with diffuse plaques (Fig. 4E). After randomly counting 88 focal Aβ plaques and 74 NPs from 5 hippocampi covering the “L/I/H” groups, 70.5% of focal plaques coincided with NPs, and 83.8% of NPs coincided with focal plaques (Fig. 4F).

**Fig. 5:**
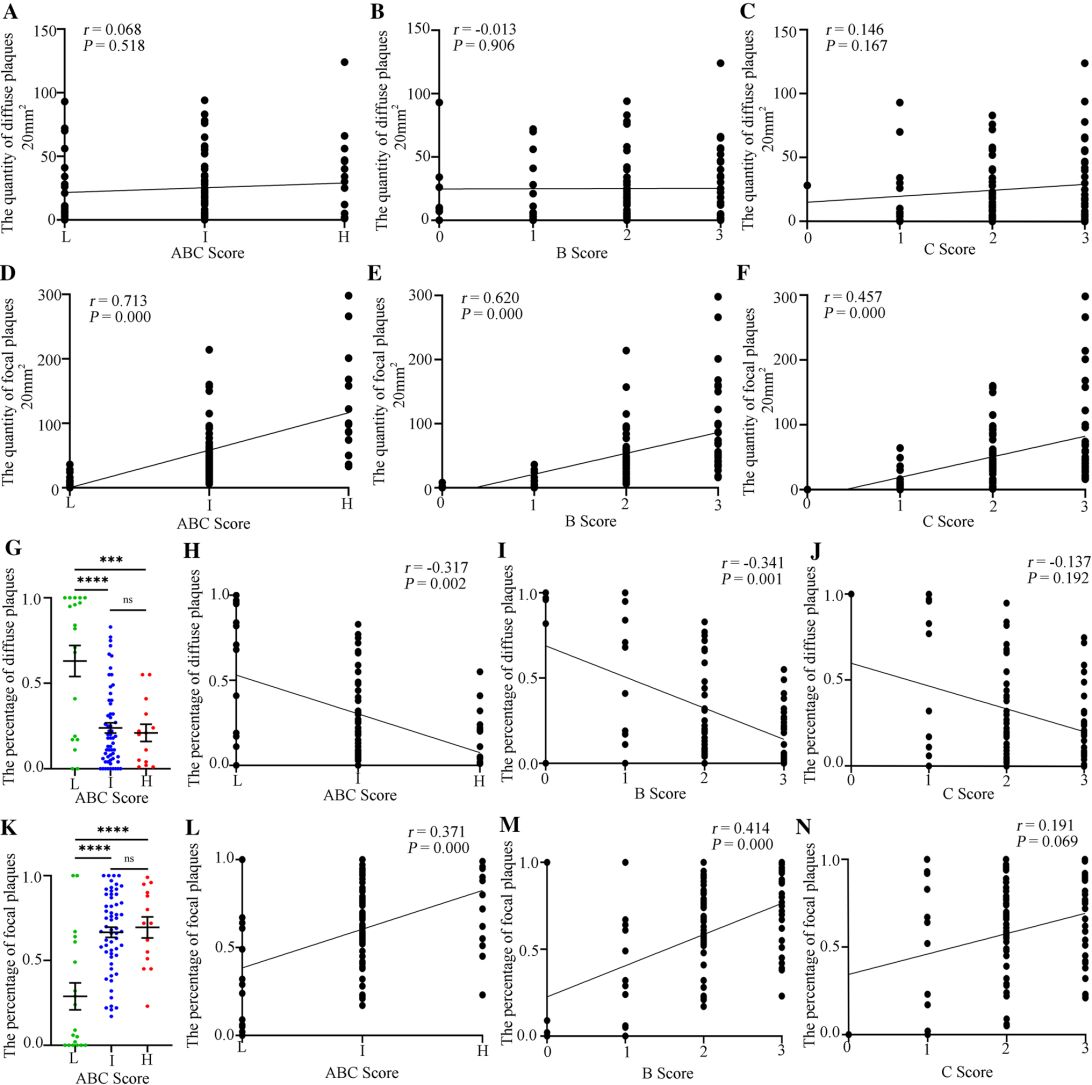
Correlations between ABC scores and different Aβ plaque forms in the hippocampus. **A** Correlation between number of diffuse Aβ plaques and ABC score. **B** Correlation between number of diffuse Aβ plaques and B score. **C** Correlation between number of diffuse Aβ plaques and C score. **D** Correlation between number of focal plaques and ABC score. **E** Correlation between number of focal plaques and B score. **F** Correlation between number of focal plaques and C score. **G** Percentage of diffuse Aβ plaques in different ABC score groups. ***P <0.001, ****P <0.0001, ns, no significant difference, by one-way ANOVA followed by Scheffe’s *post-hoc* test. **H** Correlation between ABC score and the percentage of diffuse Aβ plaques. *P* <0.05 for significance, *r* = –0.317 for negative correlation, by Spearman correlation. **I** Correlation between B score and the percentage of diffuse Aβ plaques. *P* <0.05 for significance, *r* = –0.341 for negative correlation, by Spearman correlation. **J** Correlation between C score and percentage of diffuse Aβ plaques. **K** Percentage of focal Aβ plaques in different ABC score groups. ****P <0.0001, ns, no significant difference by one-way ANOVA followed by Scheffe’s *post-hoc* test. **L** Correlation between ABC score and percentage of focal Aβ plaques. *P* <0.05 for significance, *r* = 0.371 for positive correlation by Spearman correlation. **M** Correlation between B score and percentage of focal Aβ plaques. *P* <0.05 for significance, *r* = 0.414 for positive correlation by Spearman correlation. **N** Correlation between C score and percentage of focal Aβ plaques. In (**A**–**N**), L group = 19 samples, I group = 59 samples, and H group = 14 samples. *P* <0.05 for significance, *r* >0.5 for positive correlation by Spearman correlation.

Spearman rank correlation analysis showed no significant correlation between the number of diffuse plaques and ABC score, B score, and C score (Fig. 5A–C); however, they were all moderately positive (Fig. 5D–F). It seems that focal Aβ plaques, rather than diffuse plaques, play an important role in the neuropathological changes of AD. We made further efforts to evaluate the relationship between the percentage of each plaque type and AD neuropathological changes. One-way ANOVA revealed that the percentage of diffuse plaques in the “I” and “H” groups was significantly lower than that in the “L” group (Fig. 5G). Conversely, the percentage of focal plaques in the “I” and “H” groups was significantly greater than that in the “L” group (Fig. 5K). Spearman rank correlation analysis revealed that the correlation between diffuse plaque percentage and ABC score and B score was significantly negative for low intensity (Fig. 5H, I), while the percentage of focal plaques was significantly positively correlated with both ABC score and B score (Fig. 5L, M). We did not obtain significant results between the percentage of diffuse and focal plaques and the C score (Fig. 5J, N). The above results further suggest that focal Aβ plaques, rather than diffuse plaques, participate in AD neuropathological changes.

### The Association Between Hippocampal Aβ Plaque Forms and Cognitive Dysfunction

**Fig. 6:**
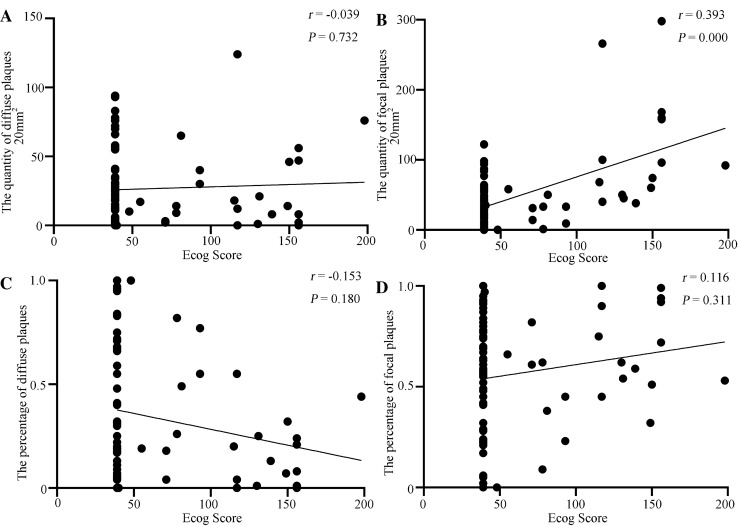
Correlations between ECog scores and the different Aβ plaque forms in the hippocampus. **A** Correlation between number of diffuse Aβ plaques and ECog score. **B** Correlation between number of focal Aβ plaques and ECog score. *P* <0.05 for significance, *r* = 0.393 for positive correlation by Spearman correlation. **C** Correlation between diffuse Aβ plaque percentage and ECog score. **D** Correlation between focal Aβ plaque percentage and ECog score.

Spearman correlation analysis was used to further confirm the relationship between different Aβ plaque forms in the hippocampus and cognitive dysfunction. The number of focal Aβ plaques, but not diffuse Aβ plaques, had a significantly positive correlation with the ECog score (Fig. 6A, B). However, the percentages of neither diffuse nor focal Aβ plaques showed significant correlation with the ECog score (Fig. 6C, D).

### The Association Between Hippocampal Aβ Plaque Forms and Neuroinflammation

**Fig. 7:**
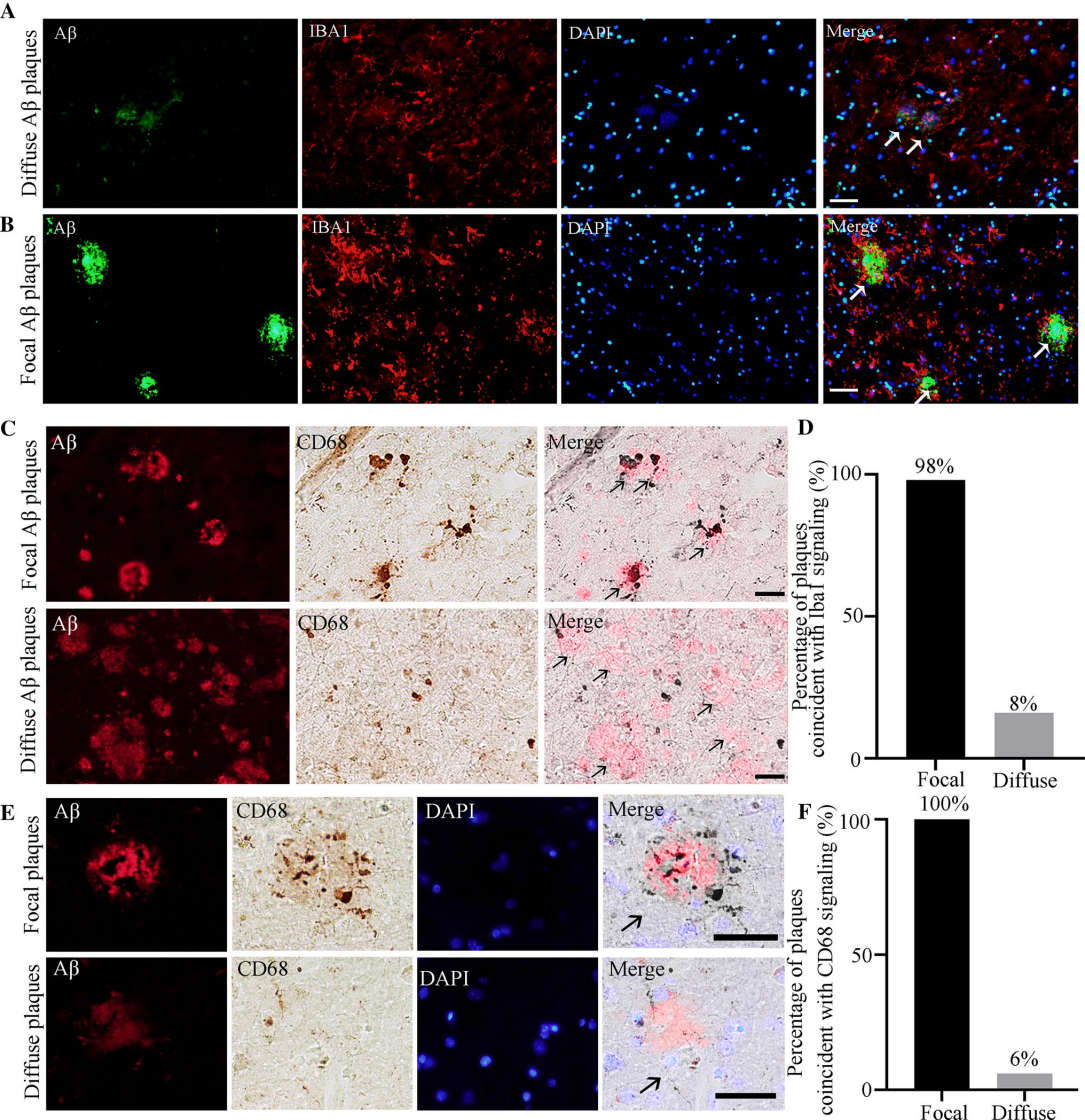
Relationships between microglial cells and the different Aβ plaque forms in the hippocampus. **A** Representative images of double immunostaining for diffuse Aβ plaques, IBA1, and DAPI. **B** Representative images of double immunostaining for focal Aβ plaques, IBA1, and DAPI. **C** Representative images of double immunostaining for different types of Aβ plaque and CD68. **E** Representative images of double immunostaining for focal Aβ plaques, CD68, and DAPI. Scale bars, 50 μm in (**A–C**) and (**E**). **D, F** Percentages of different types of plaques coincident with IBA1- (**D**) and CD68-positive (**F**) cells. *n* = 50 for each plaque type in the hippocampus from 6 human brain samples covering the “L/I/H” group.

**Fig. 8:**
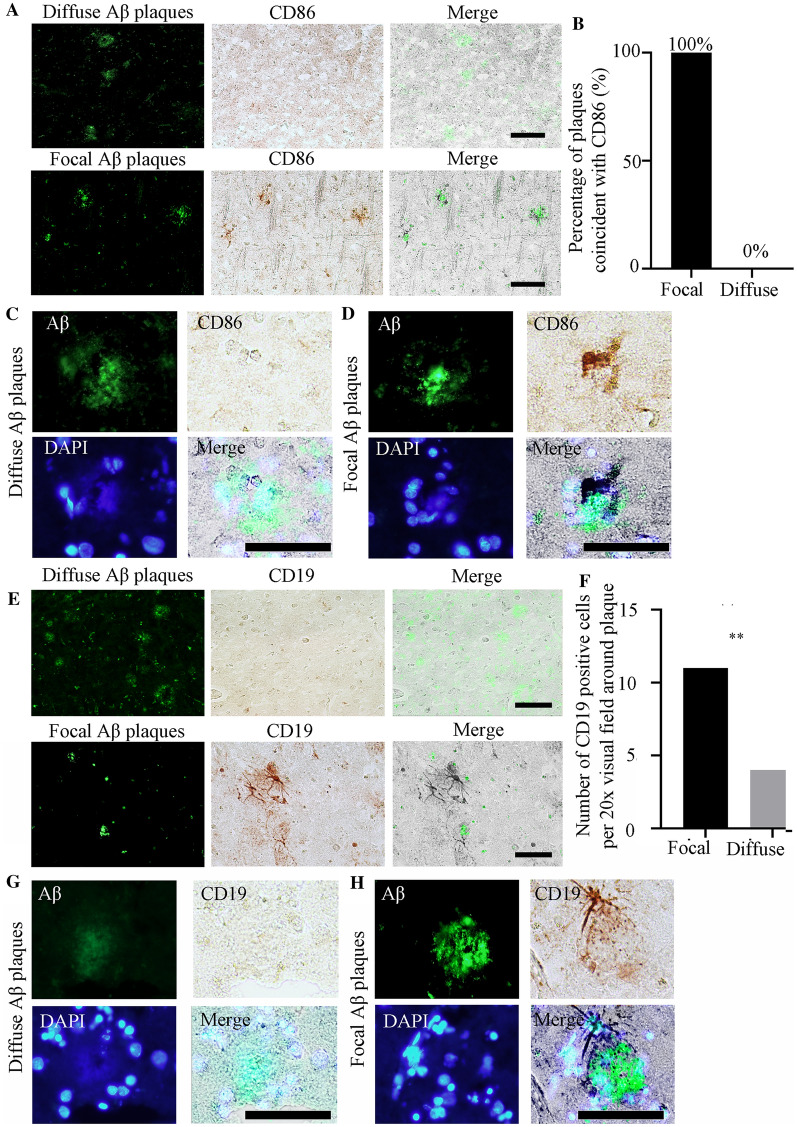
Relationships between CD86- or CD19-positive cells and Aβ plaque formation in the hippocampus. **A** Representative images of double immunostaining for different types of Aβ plaque and CD86. **B** Percentages of different types of plaques coincident with CD86-positive cells. *n* = 50 per plaque type from 6 human brain samples covering the “L/I/H” group.** C** Representative images of double immunostaining for diffuse Aβ plaques, CD86, and DAPI. **D** Representative images of double immunostaining for focal Aβ plaques, CD86, and DAPI. **E** Representative images of double immunostaining for different types of Aβ plaque and CD19. **F** Number of CD19-positive cells per 20× visual field around different types of Aβ plaque. *n* = 50 per plaque type in 6 human brain samples covering the “L/I/H” group. **G** Representative images of double immunostaining for different types of diffuse Aβ plaque, CD19, and DAPI. **H** Representative images of double immunostaining for different types of focal Aβ plaque, CD19, and DAPI. Scale bars, 50 μm in (**A**), (**C**), (**D**), (**E**), (**G**), and (**H**).

The pathological changes of AD are closely correlated with neuroinflammation [41]. Immunostaining showed that the focal Aβ plaques were surrounded by more microglia than diffuse Aβ plaques in the hippocampus (Fig. 7A, B). Fifty plaques from 6 samples covering the “L/I/H” groups for each type were randomly selected, and the statistical results showed significantly more focal plaques coincided with IBA1-positive cells than diffuse plaques (Fig. 7C). CD68 is a heavily glycosylated glycoprotein that is strongly expressed in macrophages and other mononuclear phagocytes, including microglia. The relationship between CD68-positive cells and different types of plaque was similar to that of IBA1 (Fig. 7E–F), further showing that microglia are preferentially attracted by focal rather than diffuse Aβ plaques. We also co-labeled different types of Aβ plaque with CD86 and CD19. CD86 is a transmembrane glycoprotein that is constitutively expressed on memory B cells, germinal center B cells, and macrophages. In addition, CD86 is expressed at low levels on microglia and is upregulated through interferon-γ stimulation. Our results showed that focal, but not diffuse Aβ plaques strongly overlapped with CD86-positive cells (Fig. 8A, C, D). Fifty randomly-selected focal plaques all coincided with CD86-positive cells, while no diffuse plaque coincided with CD86-positive cells (Fig. 8B). CD19 is a leukocyte differentiation antigen expressed by B cells belonging to the Ig superfamily. Double immunostaining showed that more CD19-positive cells were found near the focal plaques than the diffuse plaques (Fig. 8E–H). This demonstrated that, in addition to microglia, focal Aβ plaques also attract B cells or other inflammatory cells to a certain extent.

## Discussion

The neuropathological changes in AD are ranked along three parameters – Aβ plaque score, Braak NFT stage, and CERAD NP score – to obtain an ABC score [36]. The A score reveals the distribution of Aβ deposits in separate brain regions, and the C score reflects the density of cortical NPs [36], while the B score reveals the distribution of NFTs, which initially appear in the entorhinal cortex and then diffuse to the hippocampus and neocortex [42]. Several pieces of evidence suggest that NFTs may be the main driver of neurodegeneration in AD, and only when tau spreads from the entorhinal cortex into the neocortex can cognitive impairment be noted [43–45]. Although early studies have revealed that the accumulation of Aβ plaques enhances the formation and progression of tau pathology and NPs, it is not fully clear which specific Aβ plaque types are involved. Many studies have shown a lack of correlation between Aβ plaques and cognitive decline in AD patients [20–22]. In particular, our previous study reported that the C score had a significant correlation with the ECog score, while the A score had no significant correlation with any of the ECog domains, indicating that more comprehensive and detailed analysis of Aβ pathology was needed [22]. In the present study, we specifically assessed the relationship between morphologically different forms of Aβ plaque and the pathological changes in AD. We researched the correlation between different Aβ plaque forms in the hippocampus and ABC score, B score, or C score, and found that the number of focal plaques was positively correlated with the three scores. Moreover, the overwhelming majority of focal plaques coincided with NPs of the C score, while diffuse plaques did not. Our results showed that the more focal Aβ plaques in the hippocampus, the worse was the general AD neuropathology, while there was no relationship between the quantity of diffuse plaques and the severity of neuropathological changes in AD. Further calculating the percentages of focal and diffuse plaques, the percentage of focal plaques was still positively correlated with the ABC score and B score, and the percentage of diffuse plaques changed to become negatively correlated with these two scores. These results further suggest that focal plaques in the hippocampus may contribute to the neuropathological changes in AD. However, follow-up experiments are needed to further determine whether the amyloid plaques in older individuals and AD patients with severe pathological symptoms are larger and thus harbor larger cores. Whether human brains were accurately derived from familial or sporadic AD patients is also needed for research. Symptomatic AD follows an insidious and progressive course, which is characterized by early impairment in learning and memory, followed by later impairments in complex attention, executive function, language, visuospatial function, praxis, gnosis, and behavior and/or social abnormalities [46]. Here, we found that the number of focal Aβ plaques was positively correlated with the ECog score. Diffuse plaques had no correlation with the ECog score. Our results demonstrate that focal plaques rather than diffuse plaques in the hippocampus can predict the cognitive level to some extent.

Microglia are primary inflammatory cells in the brain. Activated microglia are characterized by morphological and functional changes, including but not limited to increased phagocytosis and increased expression of receptors, cytokines, chemokines, and additional inflammation-related molecules [47]. Reactive glial cells occur within neuritic plaques, and further studies have shown that both reactive astrocytes and microglia occur in the vicinity of Aβ plaques [35, 48]. Reactive glia and associated neuroinflammation are now regarded as playing key roles in both disease initiation and progression [35, 41, 47]. Our findings revealed that there were always a considerable number of microglia around the focal plaques in the hippocampus, while diffuse plaques had a poor ability to attract these microglia. This phenomenon indicates that focal rather than diffuse Aβ plaques in the hippocampus are strongly correlated with neuroinflammation. We also co-localized different types of Aβ plaque with CD19, a leukocyte differentiation antigen expressed by B cells, to research the relationship between Aβ plaques and B lymphocytes. As in a previous study, B lymphocytes in the central nervous system were mostly located in the meninges and cerebrospinal fluid [49–51]. Our results showed that there were also B lymphocytes in the brain parenchyma, and there were more B lymphocytes near focal plaques than diffuse plaques, suggesting the regulatory mechanism of the adaptive immune response in focal Aβ neuropathology. These findings suggest that the adaptive immune response in the central nervous system may be involved in the pathogenesis and development of AD neuropathology.

There is a view that diffuse plaques form earlier from the accumulation of amyloid-beta and then evolve over time into dense-core plaques. This model also emphasizes that extracellular amyloid is pathological and leads to inflammation. This model, however, does not adequately explain the occurrence or fate of intraneuronal amyloid, nor does it reveal any relation between the intraneuronal amyloid burden and amyloid plaques. Much experimental evidence has shown that there are different origins of amyloid plaques in the human brain. Diffuse plaques do not contain neuron-derived DNA or cytoplasmic proteins, have nothing to do with neuronal death and lysis, and do not seem to affect local axons or dendrites. Focal plaques contain the residue released by cell lysis after neuronal necrosis, and this surrounds the dense core usually containing neuronal nuclei in the form of spherical clouds. Various staining results have shown that the dense core of focal plaques has a certain resistance to the proteolysis of lysosomal enzymes released during neuronal lysis; they are proteolysis-resistant proteins. Centromeric sequences found in focal plaques represent residual DNA fragments, which are dispersed in plaques after neuronal nuclear degradation [30]. The presence of neuron-derived focal plaques can easily explain the relationship between the increase in Aβ plaques and the decrease in neurons in the cerebral cortex in AD [52]. Our study demonstrates that the number of focal plaques rather than diffuse plaques is associated with more severe cognitive impairment and inflammatory infiltration, and it is more toxic, which further supports the view of different origins. Moreover, the Aβ components of different types of plaque are different. Aβ42 is the main component of plaques, and its aggregation rate is faster than that of Aβ40 [53]. Diffuse plaques and loosely-packed focal plaque material mainly consist of filamentous Aβ42, whereas plaque cores are made of both Aβ40 and Aβ42.

The neuropathological changes in AD are associated with focal Aβ plaques rather than diffuse Aβ plaques in the hippocampus. More importantly, focal Aβ plaques rather than all forms of plaque in the hippocampus are positively correlated with recognition dysfunction. Moreover, compared to diffuse Aβ plaques, focal Aβ plaques are more associated with neuroinflammation, regardless of the innate immune response or adaptive immune response.

## Supplementary Information

Below is the link to the electronic supplementary material.

Supplementary file1 (PDF 564 kb)
