# Supplementary material for: Focal-type, but not Diffuse-type, Amyloid Beta Plaques are Correlated with Alzheimer’s Neuropathology, Cognitive Dysfunction, and Neuroinflammation in the Human Hippocampus
Source: Neurosci Bull. 2022 Aug 26;38(10):1125–38. doi: 10.1007/s12264-022-00927-5 (PMC9554074; doi:10.1007/s12264-022-00927-5)
Supplement: Supplementary file 1 — Supplementary file1 (PDF 564 kb) [file 12264_2022_927_MOESM1_ESM.pdf]

Supplemental Materials

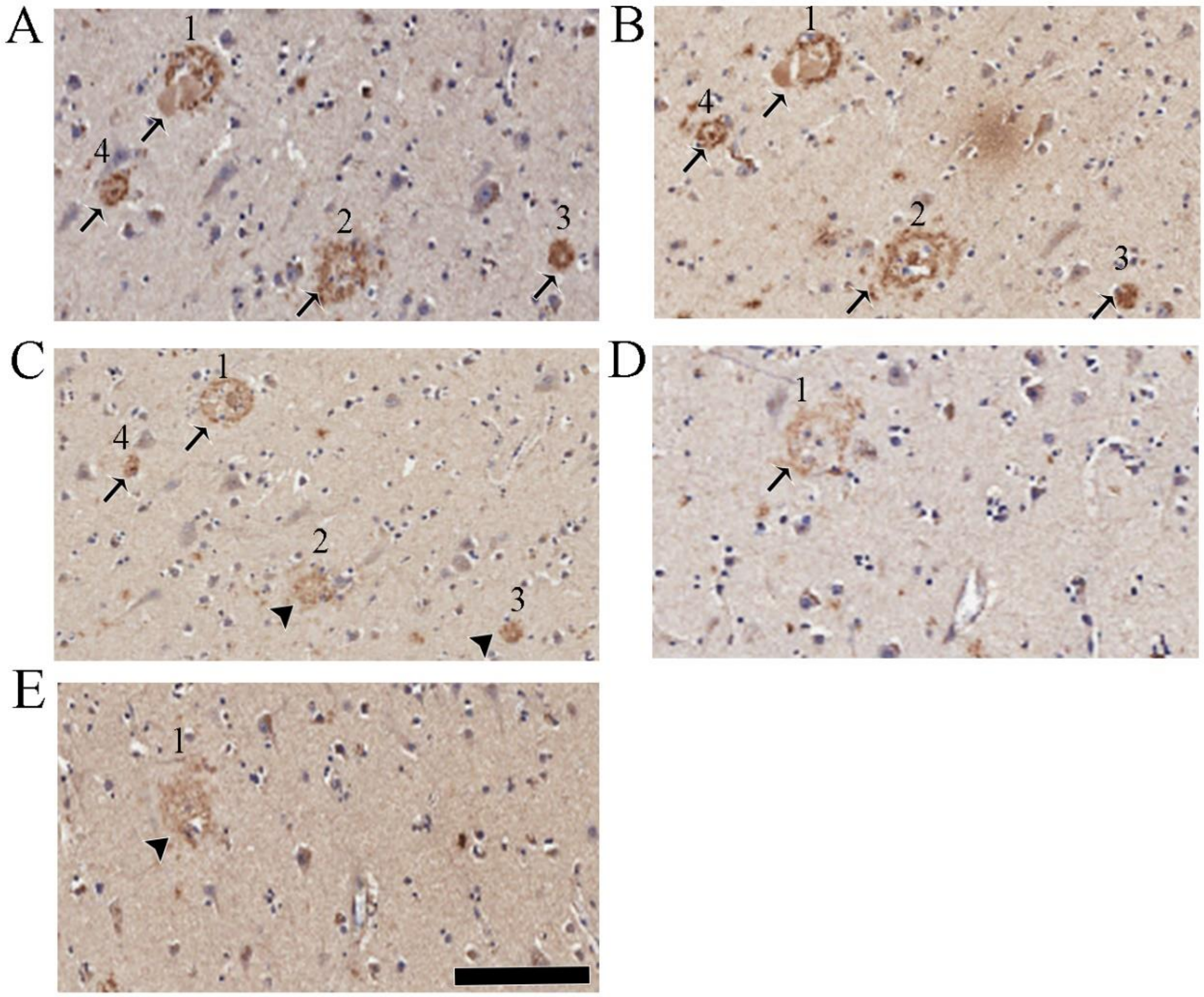

Fig. S1 Morphology and type judgment of focal Aβ plaques in continuous sections. **A**–**E** Aβ plaques numbered 1–4 were judged to be focal plaques through the overall interpretation of 5 consecutive sections (arrows, plaques determined to be focal Aβ plaques in the current section; arrowhead, plaque determined to be a diffuse Aβ plaque in the current section; scale bar, 50 μm).

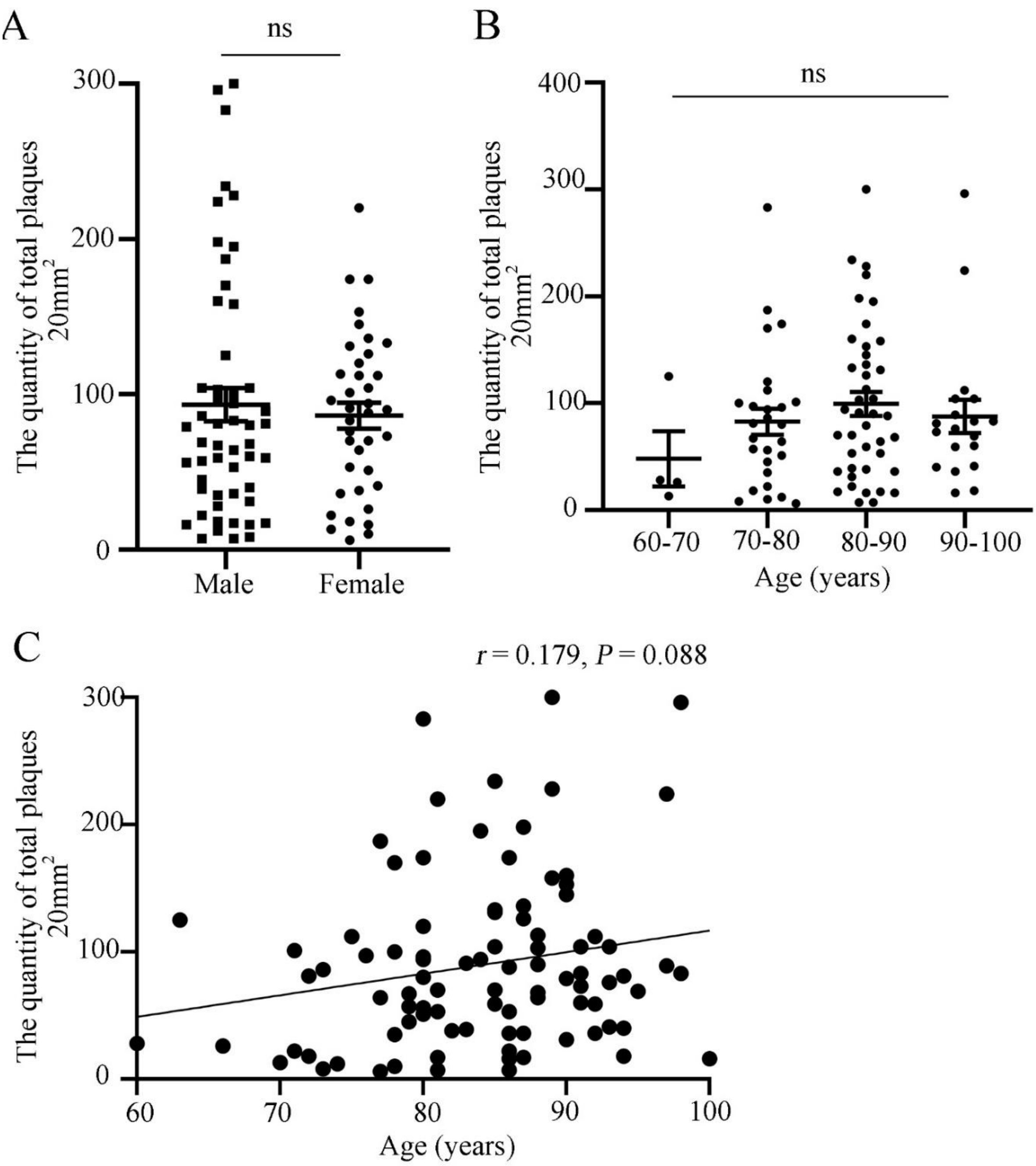

Fig. S2 The contributions of demographic factors to total Aβ plaques in the hippocampus. **A** Total numbers of Aβ plaques by sex. male = 53 samples, female = 39 samples, male group *versus* female group, ns, no significant difference by Student's *t* test. **B** Total numbers of Aβ plaques in different age groups. The age group of 60–70 years)= 4 samples, 70–80 years = 27 samples, 80–90 years = 42 samples, 90–100 years = 19 samples. ns, no significant difference by one-way ANOVA followed by Scheffe's *post-hoc* test. **C** Correlation between the total number of Aβ plaques and age. n = 92 human brain samples; P >0.05, no significant correlation by Spearman correlation.

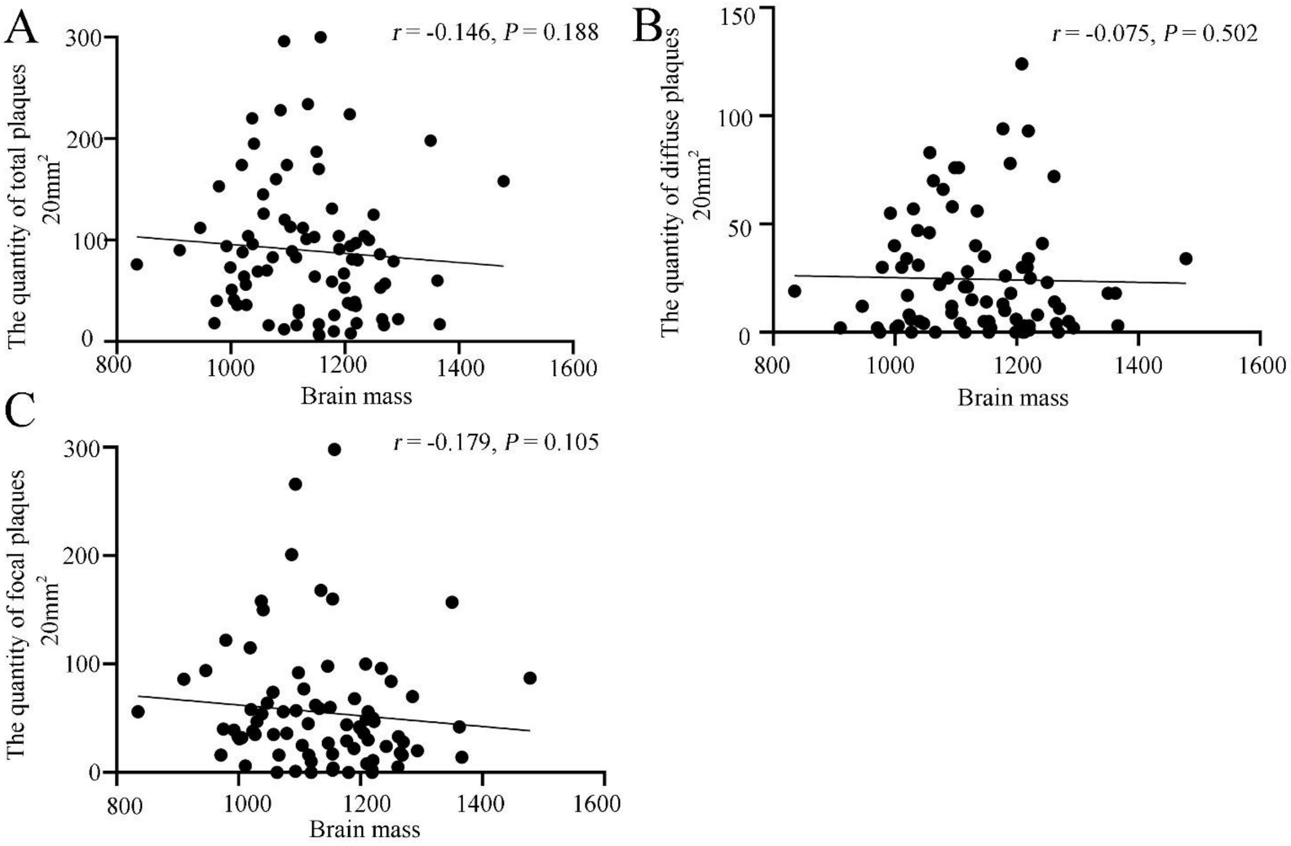

Fig. S3 Correlations between different types of Aβ plaque and human brain atrophy. **A** Correlation between the total number of Aβ plaques and human brain mass. **B** Correlation between the number of diffuse Aβ plaques and human brain mass. **C** Correlation between the number of focal Aβ plaques and human brain mass. n = 83 human brain samples, P >0.05, no significant correlation by Spearman correlation.

Table S1 Detailed information on all donors

No.	Gender	Age (years)	PMD (h)	ABC score	A score	B score	C score	Brain mass (g)	ECog score
Sample01	F	87	5	I	2	3	2	null	null
Sample02	M	88	6.5	I	2	2	2	null	null
Sample03	M	81	12	L	2	0	1	null	null
Sample04	F	85	4.5	I	2	3	3	null	81
Sample05	F	85	7	I	2	2	2	null	39
Sample06	M	85	7.5	L	3	1	2	null	39
Sample07	M	79	6.5	I	3	2	3	null	39
Sample08	M	84	5	I	2	3	2	1040	null
Sample09	M	90	18	I	2	3	3	1285	null
Sample10	F	98	69	I	2	3	2	1073	null
Sample11	M	80	57	I	2	2	3	1222	39
Sample12	M	92	16.5	I	2	2	1	1212	null
Sample13	F	86	6.3	I	3	2	3	1020	55
Sample14	F	80	18	I	2	2	2	1001	71
Sample15	M	73	18	L	2	0	1	1210	39
Sample16	M	78	8	L	2	0	1	1219	39
Sample17	F	78	20	L	2	0	1	1180	48
Sample18	F	77	21	L	2	1	1	1155	39
Sample19	F	87	43	I	2	3	2	1027	40

Sample20	M	90	5	L	2	1	2	1119	39
Sample21	M	72	22.2	H	3	3	3	1220	130
Sample22	F	83	3	I	2	3	3	1190	115
Sample23	F	80	13	I	2	2	2	1098	198
Sample24	F	81	4	L	2	1	1	1063	39
Sample25	F	91	3	H	3	3	3	999	93
Sample26	M	77	23	I	2	2	3	1150	149
Sample27	M	83	10	I	2	2	1	1217	93
Sample28	F	100	16	I	2	3	1	1115	null
Sample29	F	70	4.5	I	2	2	1	null	39
Sample30	M	86	37.5	L	2	1	2	1154	39
Sample31	M	97	26.5	H	3	3	3	1208	117
Sample32	F	86	8	I	2	2	2	1019	null
Sample33	F	84	4	I	3	2	3	992	39
Sample34	F	86	18	I	2	2	1	1011	39
Sample35	M	86	10	I	2	2	1	1268	39
Sample36	M	86	20.5	I	2	1	2	1066	39
Sample37	F	91	12	I	2	3	2	1030	39
Sample38	M	91	16.5	I	3	2	3	1114	131
Sample39	M	91	18	I	2	3	2	1362	39
Sample40	F	81	8	H	3	3	3	1037	156
Sample41	F	90	91.3	H	3	3	3	1056	150

Sample42	M	74	7.5	L	2	0	2	1093	78
Sample43	M	81	28	I	1	2	2	1366	71
Sample44	M	92	43	I	2	2	2	1177	39
Sample45	M	93	6	I	3	2	3	1234	156
Sample46	F	82	12.5	I	2	2	2	1205	39
Sample47	M	80	6	L	2	1	1	1026	39
Sample48	M	80	71.5	I	3	2	3	null	null
Sample49	M	94	9.5	I	2	3	2	975	117
Sample50	M	87	8.8	I	2	3	3	1154	null
Sample51	M	78	13.5	I	2	3	2	1154	156
Sample52	F	88	7	I	2	2	2	1023	139
Sample53	F	66	17.5	L	1	0	1	1181	39
Sample54	M	81	16	I	2	2	3	1199	39
Sample55	M	77	4	I	2	3	3	1147	39
